# Ginsenoside Rg1 alleviates acute liver injury through the induction of autophagy and suppressing NF-κB/NLRP3 inflammasome signaling pathway

**DOI:** 10.7150/ijms.50919

**Published:** 2021-01-23

**Authors:** Jinqiu Zhao, Bin He, Shujun Zhang, Wenxiang Huang, Xiaosong Li

**Affiliations:** 1 Department of Infectious Diseases, The First Affiliated Hospital of Chongqing Medical University, Chongqing, 400016, China.; 2 Department of Orthopedics, The First Affiliated Hospital of Chongqing Medical University, Chongqing, 400016, China.; 3 Clinical Molecular Medicine Testing Center, The First Affiliated Hospital of Chongqing Medical University, Chongqing, 400016, China.

**Keywords:** Ginsenoside-Rg1, acute liver injury, autophagy, NF-κB, NLRP3 inflammasome.

## Abstract

**Background:** Severe hepatitis is a common cause of chronic or acute liver disease and autophagy might play an important role in cellular response to inflammation and injury. It has been reported that Ginsenoside-Rg1 (G-Rg1) has strong hepatoprotective effects for acute liver injury, but its protective mechanisms have not yet been elucidated. This study aims to explore the detailed molecular mechanisms of G-Rg1 on acute liver injury via autophagy.

**Methods:** The role of G-Rg1 by autophagic induction was studied in the mouse model of acute liver injury which induced by carbon tetrachloride (CCl4). Liver function, inflammatory reaction and apoptosis were detected when autophagy has been inhibited by 3-MA or stimulated by RPA. MCC950 and ATP were applied to investigate the role of NLRP3 inflammasome in acute liver injury. The differential expression of NF-κB, NLRP3 inflammasome, caspase 1, caspase 3, IL-1β, IL-18, LC3-I, LC3-II, Beclin-1, PINK1 and Parkin have been detected by the quantitative real-time polymerase chain reaction (qRT-PCR) and Western blot.

**Results:** G-Rg1 could decrease ALT, AST, TNF-α, IL-1β and IL-6 in mice with CCl4-induced acute liver injury. The change of autophagy and apoptosis after the treatment of 3-MA or RPA demonstrated that the autophagy played a key role in the protective effect of G-Rg1 in acute liver injury. The enhancement of G-Rg1 promoted-autophagy resulted in the significant decrease in NF-κB, NLRP3 inflammasome, caspase 1, caspase 3, IL-1β and IL-18, which suggesting that NF-κB/NLRP3 inflammasome signaling pathway was associated with the autophagy induced by G-Rg1 in acute liver injury.

**Conclusion:** G-Rg1 ameliorated acute liver injury via the autophagy, which may be related to NF-κB/NLRP3 inflammasome signaling pathway.

## Introduction

Acute liver injury can result in liver function abnormality, and is caused by many reasons including viral infection, abuse of drugs or alcohol, ingestion of toxic substance etc 1-3. Serious liver injury may lead to liver failure or death 4, 5. Acute liver failure is an inflammation-mediated hepatocellular injury process, during which hepatocellular apoptosis and hemorrhagic will necrosis occur 6, 7. The prognosis for acute liver failure is extremely poor, and only liver transplantation may be effective to treat the end stage of acute liver failure 8.

Ginsenoside Rg1 (G-Rg1) is the most abundant ingredient of Panax ginseng, and exerts strong influence on reactive oxygen stress, angiogenesis, neuronal differentiation, and anti-inflammation 9-12. Previous studies have explored that the hepatoprotective effects of G-Rg1, and demonstrated the potential of G-Rg1 in reducing the production of proinflammatory factors such as TNF-α 13 and alleviating liver fibrosis 14. Our previous studies have found that G-Rg1 had the ability to improve liver function and inhibit caspase-dependent hepatocellular apoptosis in mouse model of acute liver failure 15.

Autophagy, a highly conserved catabolic process could prevent cell damage and promote survival in the event of energy or nutrient shortage and respond to various cytotoxic insults 16-18. It had multiple functions in cell survival, proliferation and apoptosis in mammals 19, 20. Some studies have reported that autophagy consist of several procedures including the sequestration of a region of cytosol in double-membrane compartments, the formation of autophagosomes, the fusion of autophagosomes with lysosomes and degradation dependent on lysosome 21, 22.

Given the above information, we proposed that G-Rg1 might play a protective role in acute liver injury by regulating autophagic pathway. In the mice model of carbon tetrachloride (CCl4)-induced acute liver injury, the liver function, inflammatory reaction and apoptosis were analyzed after the treatment of 3-methyladenine (3-MA) or enhanced rapamycin (RPA) in order to explore the role of autophagy in the protective effect of G-Rg1. Then qRT-PCR and Western blot were conducted to detect NF-κB, NLRP3 inflammasome and autophagic proteins for investigating the relationship between NF-κB/NLRP3 inflammasome signaling pathway and autophagy.

## Results

### G-Rg1 had strong hepatoprotective effects in mice with CCl4-induced acute liver injury

**Figure 1:**
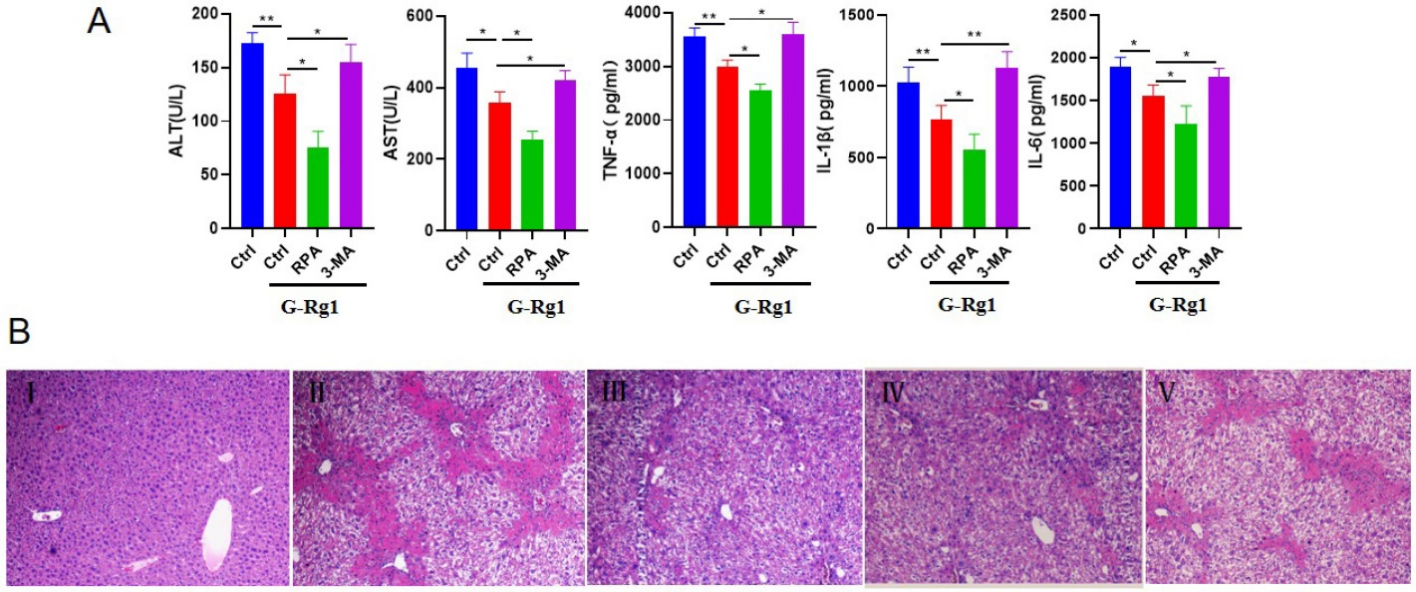
** G-Rg1 significantly improved hepatic function and inhibited inflammatory reaction in mice with CCl_4_-induced acute liver injury.** A: Serum AST, ALT, TNF-α, IL-1β, IL-6 were measured in control group, G-Rg1(4 mg/ml) control group, G-Rg1(4 mg/ml) + RPA(1 mg/kg) group and G-Rg1(4 mg/ml) + 3-MA(1 mg/kg) group have been implemented after 24 h of CCl_4_ injection (n=3). *P<0.05, **P<0.01. B: HE staining, Ⅰ: normal liver tissue, Ⅱ: CCL4 group, Ⅲ: G-Rg1 group, Ⅳ: G-Rg1+RPA (autophagy agonist) group, V: G-Rg1+3-MA (autophagy inhibitor) group.

To investigate the role of G-Rg1 in acute liver injury, the serum alanine aminotransferase (ALT) and aspartate aminotransferase (AST), TNF-α, IL-1β, IL-6 of mice were detected after 24 h injection with CCl4 (Figure 1A). The levels of ALT, AST, TNF-α, IL-1β and IL-6 in G-Rg1 treatment group were significantly reduced than those in control group, indicating that G-Rg1 treatment had the ability to improve liver function and inhibit inflammatory reaction. In addition, the autophagy agonist RPA in combination with G-Rg1 was associated with substantially reduced ALT, AST, TNF-α, IL-1β and IL-6 than G-Rg1 treatment group. Consistently, the autophagy inhibitor 3-MA addition resulted in the increase in ALT, AST, TNF-α, IL-1β and IL-6 than G-Rg1 treatment group. The HE staining also showed that G-Rg1 had strong hepatoprotective effects in mice with CCl4-induced acute liver injury (Figure 1B). The above results suggested that autophagy may play an important role in the protective effect of G-Rg1 in acute liver injury.

### G-Rg1 could promote hepatocyte autophagy in mice with CCl4-induced acute liver injury

**Figure 2:**
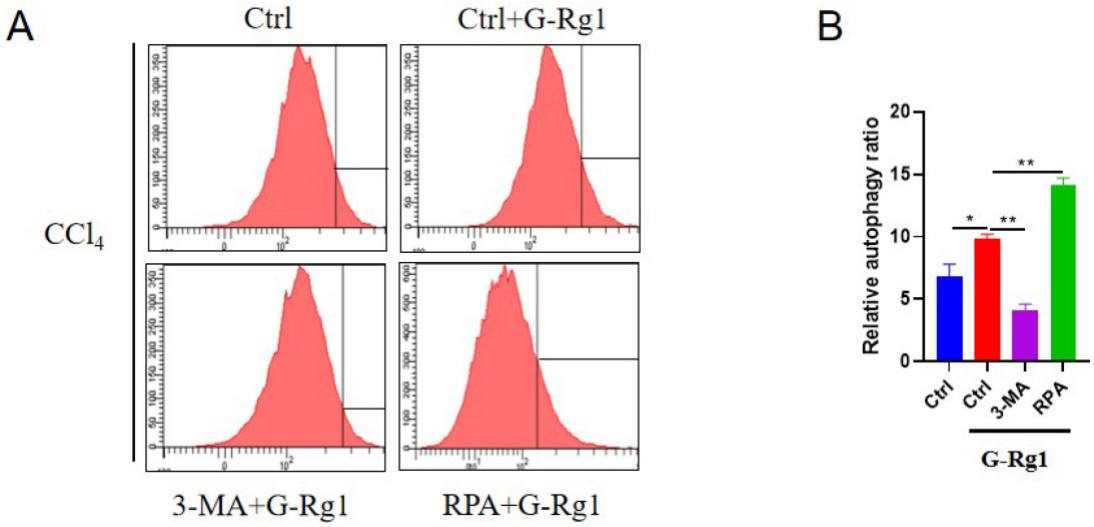
** G-Rg1 promoted autophagy for acute liver injury *in vivo*.** The cells were subjected to flow cytometry for autophagy detection after 24 h of treatment in control group, G-Rg1(4 mg/ml) control group, G-Rg1(4 mg/ml) + 3-MA(1 mg/kg) group and G-Rg1(4 mg/ml) + RPA(1 mg/kg) group (n=3).

In order to explore the mechanisms of G-Rg1 on acute liver injury in this study, then, the further research on protective effect of G-Rg1 for acute liver injury have been implemented. G-Rg1 could regulate autophagy in mice that had been exposed to CCl4 for 24 h (Figure 2). Flow cytometry showed that G-Rg1 could promote autophagy significantly than the control group, and the autophagy agonist RPA in combination with G-Rg1 could further improve autophagy. The autophagy inhibitor 3-MA plus G-Rg1 could lead to lower levels of autophagy than that in G-Rg1 treatment group which revealed that the protective effect of G-Rg1 in acute liver injury may via the induction of autophagy.

### G-Rg1 could suppress hepatocyte apoptosis in mice with CCl4-induced acute liver injury

**Figure 3:**
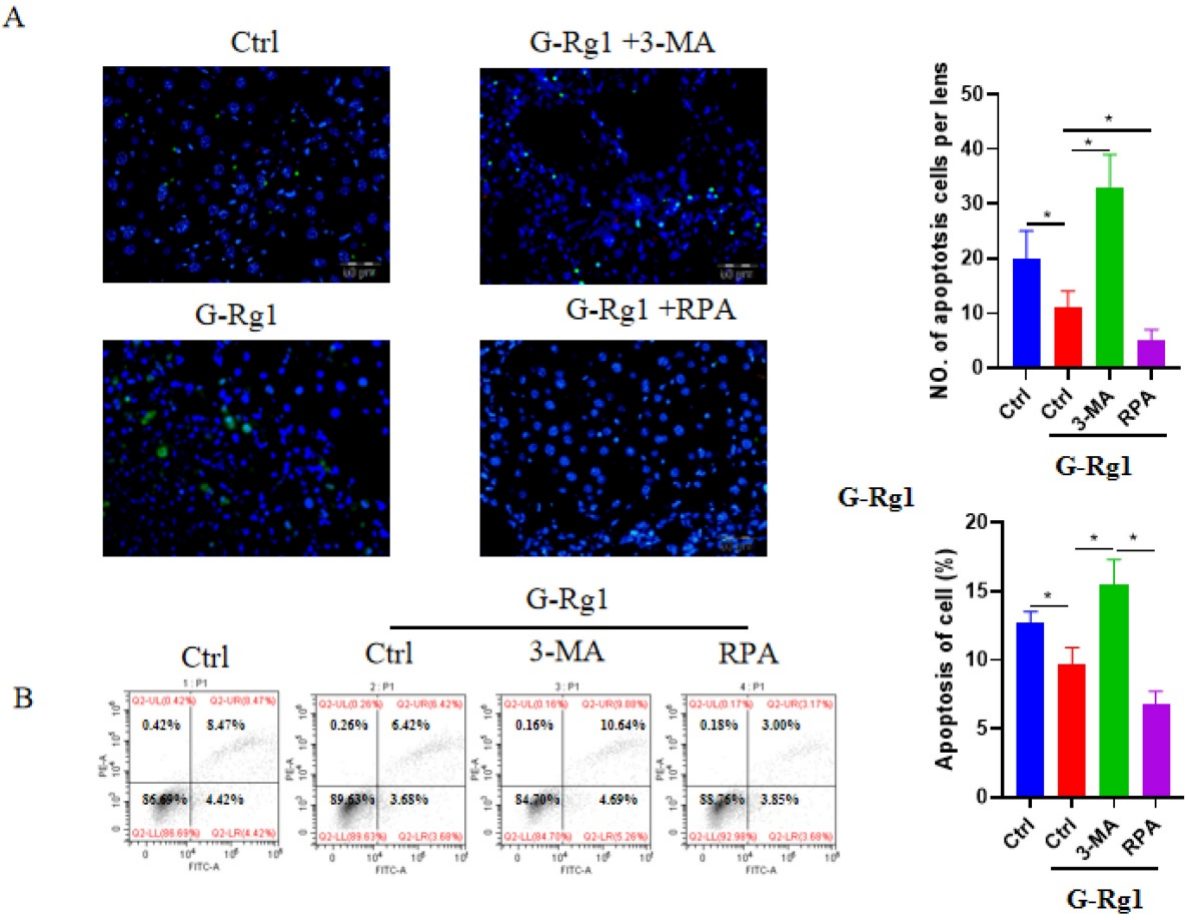
** G-Rg1 suppressed apoptosis in mice with CCl_4_-induced acute liver injury through autophagic induction.** (A) Apoptotic cells were stained with a TUNEL apoptosis detection kit in control group, G-Rg1(4 mg/ml) control group, G-Rg1(4 mg/ml) + RPA(1 mg/kg) group and G-Rg1(4 mg/ml) + 3-MA(1 mg/kg) group at 24 h after the CCl_4_ injection. All of the images were obtained on an inverted fluorescence microscope. (B) Flow cytometry was also performed to detect the apoptotic hepatocytes (n=3). *P<0.05.

The apoptotic hepatocytes were detected by TUNEL staining and flow cytometry at 24 h after the CCl4 injection (Figure 3). The TUNEL staining revealed that G-Rg1 treatment group could reduce the cell apoptosis significantly more than control group, and the addition of autophagy agonist RPA was able to further decrease apoptotic hepatocytes. In contrast, the combination of autophagy inhibitor 3-MA with G-Rg1 showed more apoptotic hepatocytes than G-Rg1 treatment group. These results were consistent with those outcomes of flow cytometry apart from that, combining G-Rg1 with autophagy inhibitor 3-MA resulted in more apoptotic hepatocytes than G-Rg1 plus autophagy agonist RPA, but demonstrated no statistical difference when compared to G-Rg1 treatment group. These also confirmed that G-Rg1 and autophagy activation alleviated CCl4-induced acute liver injury. These studies also confirmed that G-Rg1 could alleviate CCl4-induced acute liver injury may via autophagy activation.

### Autophagy and NLRP3 inflammasome had important roles in regulating acute liver injury

**Figure 4:**
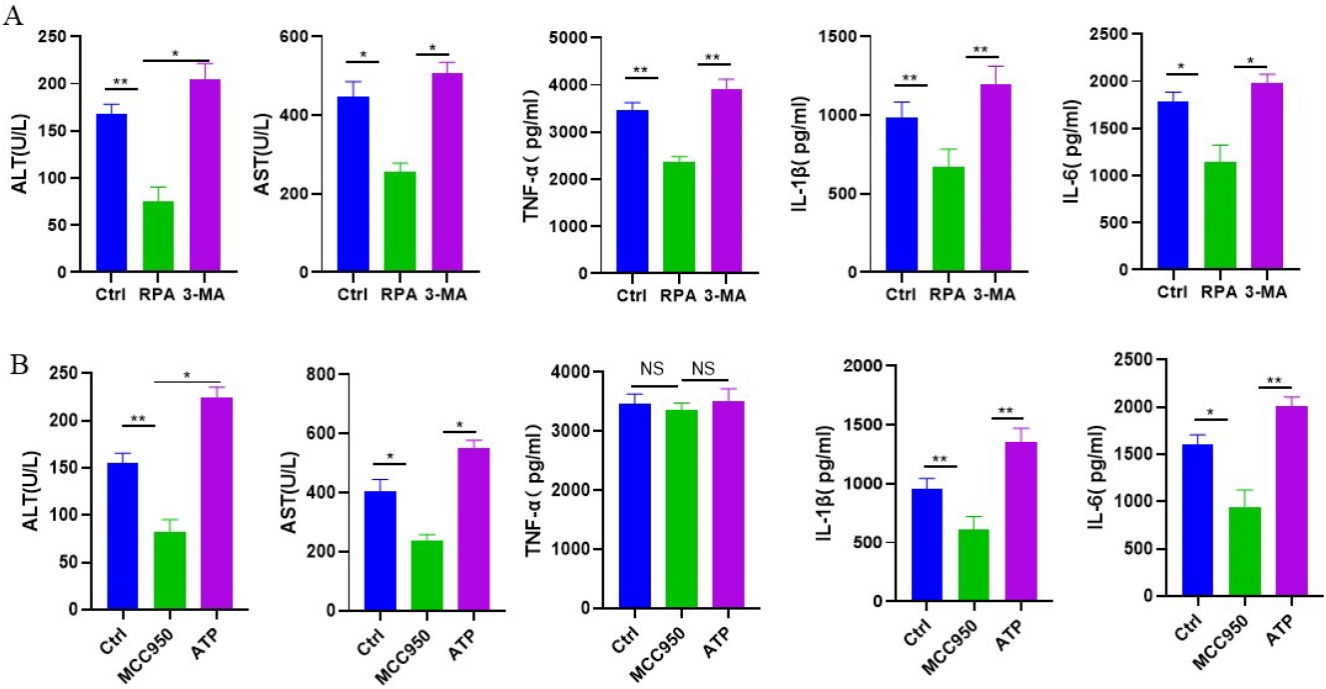
** The roles of autophagy and NLRP3 inflammasome in mice with CCl_4_-induced acute liver injury.** (A) Serum AST, ALT, TNF-α, IL-1β, IL-6 were measured in control group, RPA group and 3-MA group 24 h after the CCl_4_ injection. (B) Serum AST, ALT, TNF-α, IL-1β, IL-6 were measured in control group, MCC950 group and ATP group 24 h after the CCl_4_ injection (n=3). *P<0.05, **P<0.01, NS, no significant.

We then evaluated whether autophagy and NLRP3 inflammasome could play an important role in mice with CCl4-induced acute liver injury. The serum ALT, AST, TNF-α, IL-1β and IL-6 were examined at 24 h after the CCl4 injection. When compared to the control intervention, the autophagy agonist RPA could reduce the levels of ALT, AST, TNF-α, IL-1β and IL-6, while the autophagy inhibitor 3-MA showed significantly higher ALT, AST, TNF-α, IL-1β and IL-6 than autophagy agonist RPA (Figure 4A). These results suggested that the induction of autophagy improved liver function and inhibited inflammatory reaction in CCl4-induced acute liver injury. In addition, the NLRP3 inflammasome inhibitor MCC950 could reduce the levels of ALT, AST, IL-1β and IL-6 than the control intervention, but had no obvious effect on TNF-α. The NLRP3 inflammasome agonist ATP could increase the levels of ALT, AST, IL-1β and IL-6 than the NLRP3 inflammasome inhibitor MCC950, but there was no statistical difference in TNF-α between two groups (Figure 4B). Above studies implied that NLRP3 inflammasome activation might aggravate CCl4-induced acute liver injury.

### G-Rg1 promoted autophagy through the NF-κB/NLRP3 inflammasome signaling pathway

**Figure 5:**
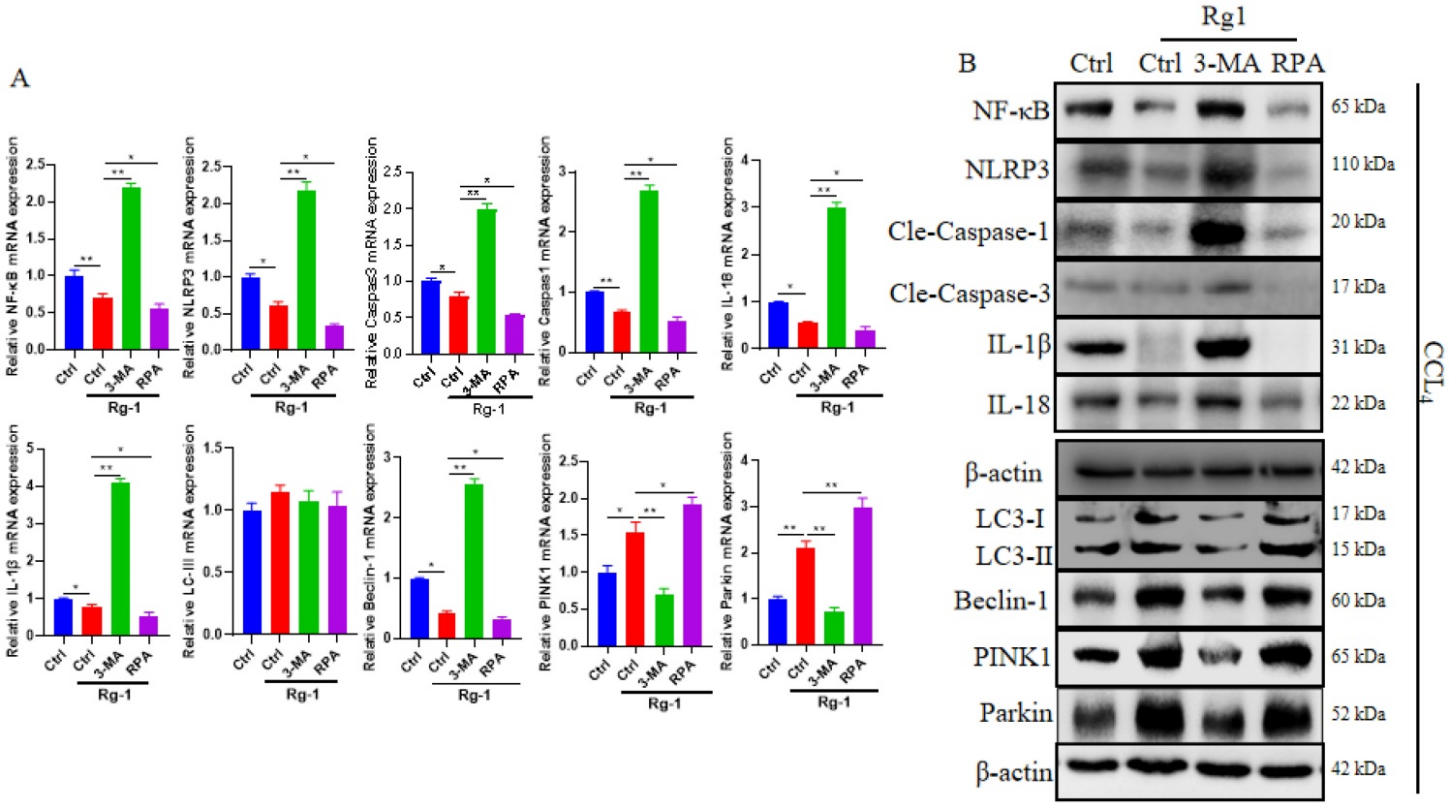
**G-Rg1-promoted autophagy was associated with the NF-κB/NLRP3 inflammasome signaling pathway.** (A) The mRNA levels of NF-κB, NLRP3 inflammasome, *cleaved* caspase 1, *cleaved* caspase 3, IL-18, IL-1β, LC3-II, Beclin-1, PINK1 and Parkin were tested by qRT-PCR. (B) Protein levels of NF-κB, NLRP3 inflammasome, caspase 1, caspase 3, IL-1β, IL-18, LC3-I, LC3-II, Beclin-1, PINK1, Parkin were tested by Western blot (n=3). *P<0.05, **P<0.01, NS, no significant.

Our results revealed that NLRP3 inflammasome significantly increased the inflammatory reactions and reduced liver function in mice with CCl4-induced acute liver injury. Our previous studies have reported that G-Rg1 had the capability to decrease the expression of NF-κB in acute liver injury. G-Rg1 treatment group could reduce the expression of NF-κB, NLRP3 inflammasome, caspase 1, caspase 3, IL-1β and IL-18, but increase the level of LC3-II, PINK1 and Parkin than the control group. The results were consistent with those when comparing G-Rg1 plus autophagy agonist RPA with G-Rg1 treatment group, and comparing G-Rg1 treatment group with G-Rg1 plus autophagy inhibitor 3-MA (Figure 5A). These outcomes were also confirmed by Western blot in terms of NF-κB, NLRP3 inflammasome, caspase 1, caspase 3, IL-1β, IL-18, LC3-I, LC3-II, PINK1 and Parkin in each group (Figure 5B). In addition, G-Rg1 plus autophagy agonist RPA showed higer levels of Beclin-1 than G-Rg1 plus autophagy inhibitor 3-MA, which was consistent with that when comparing G-Rg1 with G-Rg1 plus autophagy inhibitor 3-MA (Figure 5). These results indicated that G-Rg1-triggered autophagy could result in the reduction of NF-κB and NLRP3 inflammasome and the inhibition of G-Rg1-triggered autophagy was associated with the increase in NF-κB and NLRP3 inflammasome, which suggesting that NF-κB/NLRP3 inflammasome signaling pathway might participate in the G-Rg1-promoted autophagy in mice with CCl4-induced acute liver injury.

## Discussion

G-Rg1 exerts strong hepatoprotective properties in the animal models of liver injury, but the protective mechanism needs to be further explored. In this study, we found that G-Rg1 could improve liver function, inhibit inflammatory reaction and hepatocyte apoptosis through promoting autophagic activity in mice with CCl4-induced acute liver injury.

G-Rg1 is the primary pharmacologicallyactive compound in Panax ginseng 23, 24. The trophic and protective effects of G-Rg1 have been reported in angiogenesis 9, neuroprotection 10, 24, progenitor cell proliferation 11, 12 and inhibition of renal fibrosis 25. G‑Rg1 also possesses hepatoprotective effects in several liver injury models including ischemia/reperfusion injury, lipopolysaccharide/ D-galactosamine‑induced acute liver failure, hepatic fibrosis and alcoholic liver disease 26-29. In our previous study, G-Rg1 could significantly reduce liver damage in a murine model with CCl4-induced acute liver failure through inhibiting TNF-α-induced and caspase-dependent hepatocellular apoptosis 15.

Several trials have reported the effects of G-Rg1 on the autophagy, but they showed different outcomes in certain situations. For example, in the cardiomyocytes hypoxia/reoxygenation model, G-Rg1 could inhibite the autophagy and apoptosis in the H9c2 cardiomyocytes 30. Another trial have reported that G-Rg1 could improve the survival rate and ameliorated cognitive impairments partially through regulating cerebral inflammation and apoptosis which might possibly via suppressing the non-canonical beclin 1-independent autophagy pathway 31. In contrast, G-Rg1 was effective to induce the autophagic flux and attenuate serum deprivation-induced apoptosis in Raw264.7 macrophages 32. The inhibition of apoptosis and promotion of autophagy were also observed in H9c2 cells after G-Rg1 treatment 33.

To the best of our knowledge, this is the first research to explore the effect of G-Rg1 on autophagy in CCl4-induced acute liver injury. Our results showed that the activation of autophagy has important protective effects in CCl4-induced acute liver injury, and G-Rg1 showed the significant capability to promote autophagy and suppress hepatocyte apoptosis in the mice model. Additionally, this research revealed that NLRP3 inflammasome may play an important role in the aggravation of liver function and the elevation of inflammatory responses. In our previous study, G-Rg1 treatment could significantly decrease the expression of NF-κB p65 DNA and NF-κB p65 protein in the mouse model of CCl4-induced acute liver failure 15. G-Rg1 could protect liver function against hepatic ischemia/reperfusion injury, CCl4-induced liver injury and hepatic fibrosis via inhibiting NF-κB activation and preventing inflammation 34-38.

NF-κB/NLRP3 inflammasome signaling pathway has participated in the regulation of various diseases. When mesangial cells (MCs) cultured under high- and low-glucose conditions, the lincRNA-Gm4419 might regulate the inflammation, fibrosis and proliferation through NF-κB/NLRP3 inflammasome signaling pathway, which provided new insights into the progression of diabetic nephropathy 39. NF-κB/NLRP3 inflammasome-mediated inflammation had the key role in the dysfunction of human umbilical vein endothelial cell in the high glucose condition 40. In addition, NF-κB/NLRP3 inflammasomes pathway was associated with the atherosclerotic lesions, and artemisinin might reduce atherosclerotic lesions by suppression of inflammatory reaction via the adenosine 5'-monophosphate activated protein kinase (AMPK)/NF-κB/NLRP3 inflammasomes signalling in macrophages 41.

There were NF-κB-binding sites in the NLRP3 inflammasome promoter, and NF-κB p50 have been reported to be bound to the NLRP3 inflammasome promoter 39. Furthermore, G-Rg1-triggered autophagy was associated with the reduction in NF-κB and NLRP3 inflammasome. Consistently, the inhibition of G-Rg1-triggered autophagy was associated with the increase in NF-κB and NLRP3 inflammasome. These studies suggested that G-Rg1-triggered autophagy might induce the protective effect in CCl4-induced acute liver injury through suppressing NF-κB/NLRP3 inflammasome signaling pathway. In turn, NF-κB was known as an important negative regulator of autophagy 42, 43.

## Conclusion

G-Rg1 ameliorated acute liver injury via the autophagy, which may be related to NF-κB/NLRP3 inflammasome signaling pathway.

## Material and Methods

### Animal experiments

Male C57BL/6 mice (6-8 weeks) were provided by the Laboratory Animal Center of Chongqing Medical University. All of the animals were placed in a specific pathogen-free environment and all surgical procedures and care administered to the animals were approved by the institutional ethic committee. The mice received the intraperitoneal injection of a mixture of carbon tetrachloride (CCl4, 50%) and oil (50%) at a dose of 2 ml/kg body weight. They were scarified after the CCl4 injection for 24 h. 0.2 ml G-Rg1 (4 mg/ml, Jicui Biotechnology Co., Ltd., Yunnan, China) was given after the CCl4 injection.

The suppression of autophagy was achieved by the tail vein injection of 3-methyladenine (3-MA, 1 mg/kg, Sigma) 2 h before CCl4 exposure, while intraperitoneal injection of autophagy agonist rapamycin (RPA, 1 mg/kg, Sigma) was given 0.5 h before CCl4 exposure. Intraperitoneal injections of MCC950 (10 mg/kg, MedChem Express, Shanghai, China) or 0.3 ml ATP (100 Mm, Santa Cruz Biotechnology) was used to inhibit or activate NLRP3 inflammasome.

### Enzyme-linked immunosorbent assay (ELISA)

ELISA kits specific for mouse ALT, AST, TNF-α, IL-1β and IL-6 (Cloud-Clone Corp. Wuhan, China) were used to measure their serum levels according to the manufacturer's instructions.

### Western blot analysis

After the designated treatments were performed, liver tissues and cell pellets were lysed with RIPA buffer supplemented with protease inhibitors. Total proteins were separated via 12% SDS-polyacrylamide gel electrophoresis (PAGE) and transferred to polyvinylidene difluoride (PVDF) membranes. The membranes were incubated overnight with rabbit antibodies against NLRP3 (Zymed, South San Francisco, CA, USA), cleaved caspase 1, cleaved caspase 3 (BD PharMingen, San Diego, California, USA), IL-1β (R&D Systems, USA), IL-18 (Santa Cruz Biotechnology Inc., Santa Cruz,California, USA), LC3-I, LC3-II (Sigma, USA), NF-κB, Beclin-1, PINK1, Parkin and β-actin primary antibodies (Cell Signaling Technology, Danvers, MA, USA) at 4°C. Then, the membranes were treated with a horseradish peroxidase-conjugated goat anti-rabbit secondary antibody (Cell Signaling, CA, USA) and developed with a chemiluminescent substrate (Thermo Fisher Scientific, Rockford, IL, USA). Densitometry analysis was performed using ImageJ software, and the relative levels of protein in each group were normalized to the loading control.

### Quantitative real-time polymerase chain reaction (qRT-PCR)

Total RNA was extracted using TRIzol reagent (Invitrogen, NY, USA) according to the manufacturer's instructions. First-strand cDNA was synthesized from RNA (Superscript III cDNA Synthesis Kit, Invitrogen), and the mRNA levels were estimated by QPCR using a SYBR Green PCR Kit (Invitrogen, NY, USA) with a real-time PCR system (ABI PRISM 7300, MA, USA). The relative quantity of the cycle threshold value was normalized to an internal primer.

### Flow cytometry

The percentage of autophagy was analyzed using flow cytometry with acridine orange dyeing. Cells were stained with acridine orange (10 ug/ml) (Invitrogen, Carlsbad, CA, USA) and analyzed using flow cytometry for autophagy. Changes in acridine orange positive population were determined.

500 ul binding buffer solution was added to the cell pellet and pipette the cell solution to the microtube that covered by black-paper. Then 2 ul Annexin V-FITC, 5 ul Propidium Iodide were added, followed by 5 min culture at room temperature. Apoptotic cells were then analyzed using flow cytometry.

### Fluorescence microscopy

Apoptotic cells in liver sections were stained by the TUNEL apoptosis detection kit (KeyGEN BioTECH, Nanjing, China). Nuclei were stained with 49,6-diamino-2-phenylindole (DAPI, 1 μg/ml). All images were obtained with an inverted fluorescence microscope (Nikon Eclipse E800, Tokyo, Japan).

### Statistical analysis

All data are expressed as the mean ± standard deviation (SD). Each test was performed in triplicate for each sample. One-way analysis of variance (ANOVA) followed by the post hoc LSD test was performed to compare differences among multiple groups. P < 0.05 was considered statistically significant. All data were analyzed using SPSS 11.5 software.
